# Self-stratifying microbial fuel cell: The importance of the cathode electrode immersion height

**DOI:** 10.1016/j.ijhydene.2018.07.033

**Published:** 2019-02-15

**Authors:** Xavier Alexis Walter, Carlo Santoro, John Greenman, Ioannis Ieropoulos

**Affiliations:** Bristol BioEnergy Centre, Bristol Robotics Laboratory, T-Block, Frenchay Campus, University of the West of England (UWE), Bristol, BS16 1QY, United Kingdom

**Keywords:** Bioenergy, Microbial fuel cell, Urine, Membrane-less

## Abstract

Power generation of bioelectrochemical systems (BESs) is a very important electrochemical parameter to consider particularly when the output has to be harvested for practical applications. This work studies the effect of cathode immersion on the performance of a self-stratified membraneless microbial fuel cell (SSM-MFC) fuelled with human urine. Four different electrolyte immersion heights, i.e. 14, 24, 34 and fully submerged were considered. The SSM-MFC performance improved with increased immersion up to 34. The output dropped drastically when the cathode was fully submerged with the conditions becoming fully anaerobic. SSM-MFC with 34 submerged cathode had a maximum power output of 3.0 mW followed by 2.4 mW, 2.0 mW, and 0.2 mW for the 24, 14 and fully submerged conditions. Durability tests were run on the best performing SSM-MFC with 34 cathode immersed and showed an additional increase in the electrochemical output by 17% from 3.0 mW to 3.5 mW. The analysis performed on the anode and cathode separately demonstrated the stability in the cathode behaviour and in parallel an improvement in the anodic performance during one month of investigation.

## Highlights

• Different cathode immersion depths were tested in membraneless microbial fuel cells.
• The performance and immersion depth are positively correlated.
• Completely submerged cathodes severely decrease performance.
• Stable operation for 1 month.

## Introduction

Through the metabolic activity of anaerobic electro-active respiring microorganisms, a microbial fuel cell (MFC) converts reduced organic matter (i.e. chemical energy) into electricity [1], [2]. In MFCs, microorganisms employ an anode, i.e. a solid electrode, as the terminal electron acceptor of their electroactive anaerobic [3]. This anaerobic respiration of organic matters releases smaller organic molecules, protons and CO_2_ into the electrolyte. Whilst the protons move from the anode to the cathode, the electrons pass through an external circuit before arriving at the cathode. At the cathode, the protons and electrons react through a reduction reaction with an oxidant, thus producing current (electrons flow). Several oxidants were considered for the cathode reduction reaction [4] but oxygen is preferred and the most used due to its intrinsic high reduction potential, naturally availability at practically no cost.

In addition to the conversion of chemical energy into electrical energy, the MFC technology is of interest because it can treat organic waste from various sources [5], [6], [7], with comparable removal rates to the industry sector of wastewater management [8] without spending large amount of energy compared to the existing wastewater treatment processes that are energy extensive. Due to its capacity of accepting a wide range of organic fuel, this technology could be deployed in numerous types of environment, such as remote and off-grid areas (e.g. refugee camps, small villages) [9], or the heart of modern society (e.g. wastewater treatment plans, public toilets). Several developments have been pursued in recent years on the anode and cathode electrode and on the MFC design in order to increase the power generation that despite has increased few fold still remain low [10], [11], [12]. Considering the anode electrode, several novel materials have been investigated [13], [14], [15]. Those materials have to possess characteristics such as electrical conductivity, resistance to corrosion, mechanical strength and high surface area [14]. It is important also to consider the cost factor that in a low-power producing technology has to be taken into serious account for scaling up the technology.

These features need to match the final purpose of enhancing the interaction between electroactive bacteria and the electrode and also allow the flow of organics and nutrients within the matrix. The most suitable materials that fulfil all the previously mentioned features are mainly carbonaceous materials or metallic materials [16], [17], [18], [19]. On the other hand, the oxygen reduction reaction (ORR) is often the limiting reaction occurring in MFCs due to its sluggish kinetic [20], [21]. This is due to the neutral (or circumneutral) environment containing low concentration of H^+^ and OH^−^, both being reactants of the ORR following the acidic or alkaline pathway [22], [23], [24]. In order to accelerate the ORR, a catalyst is usually used [25], [26], [27]. Three main types of catalyst are used and categorised into: i) platinum group metal (PGM) group, ii) platinum group metal-free (PGM-free) and iii) carbonaceous based [25], [26], [27]. Whilst platinum-based materials were initially adopted as cathode catalysts, low durability in polluted environments and very high cost hinder its large utilization [28], [29]. Recently PGM-free catalysts based on transition metal (e.g. Fe, Mn, Co and Ni [30], [31]) were investigated showing high performance and durability in long run experiments. Unfortunately, the production cost still remains high [32] and the commercialisation of the product in large scale is still to be implemented.

Mainly due to their lower costs, despite lower electrochemical performance, carbonaceous-based catalysts are preferred to PGM and PGM-free and highly utilised for MFCs cathodes [27], [33], [34]. Among the carbonaceous-based catalyst, the most adopted is activated carbon (AC) since it is commercially available at relatively low cost and in large quantities. AC performance in neutral media were superior to carbon black [35] and show high durability in long-term operations [36], [37]. Furthermore, AC mixed with PTFE (polytetrafluoroethylene) and pasted on a stainless steel mesh is, at the current stage, the catalyst that presents (i) a good performance to cost ratio, (ii) a simple production process and (iii) a good stability over time (i.e. mechanical and chemical resistance) [34], [38].

Considering MFC design for practical implementation, usable power levels and increased treatment efficiency are reached when numerous MFCs are assembled in stack or cascade [39], [40]. Assembling MFC stacks implies finding equilibrium between unit size, design simplicity and cost. Recently a new concept of self-stratifying membraneless MFC (SSM-MFC) was introduced. This new design not only answer these requirements, but also allows size-scalability with negligible power density losses [9], [41], [42]. The SSM-MFCs have a plurality of cathodes placed in the upper layers of the electrolyte (i.e. urine) and a plurality of anodes placed in the lower layers, a concept already employed in wastewater treatment [43]. In this work, AC-based cathodes were investigated in SSM-MFC fed with urine as organic source fuel. The effect of the cathode immersion height on the electrochemical output and the chemical oxygen demand (COD) degradation was studied. The cathodes performance was initially examined in “clean” media and then in operating SSM-MFCs. Polarisation and power curves were recorded after steady state was reached and after additional 30 days in order to study the durability in long-terms performances.

## Material and methods

### Reactors construction and operation

**Fig. 1:**
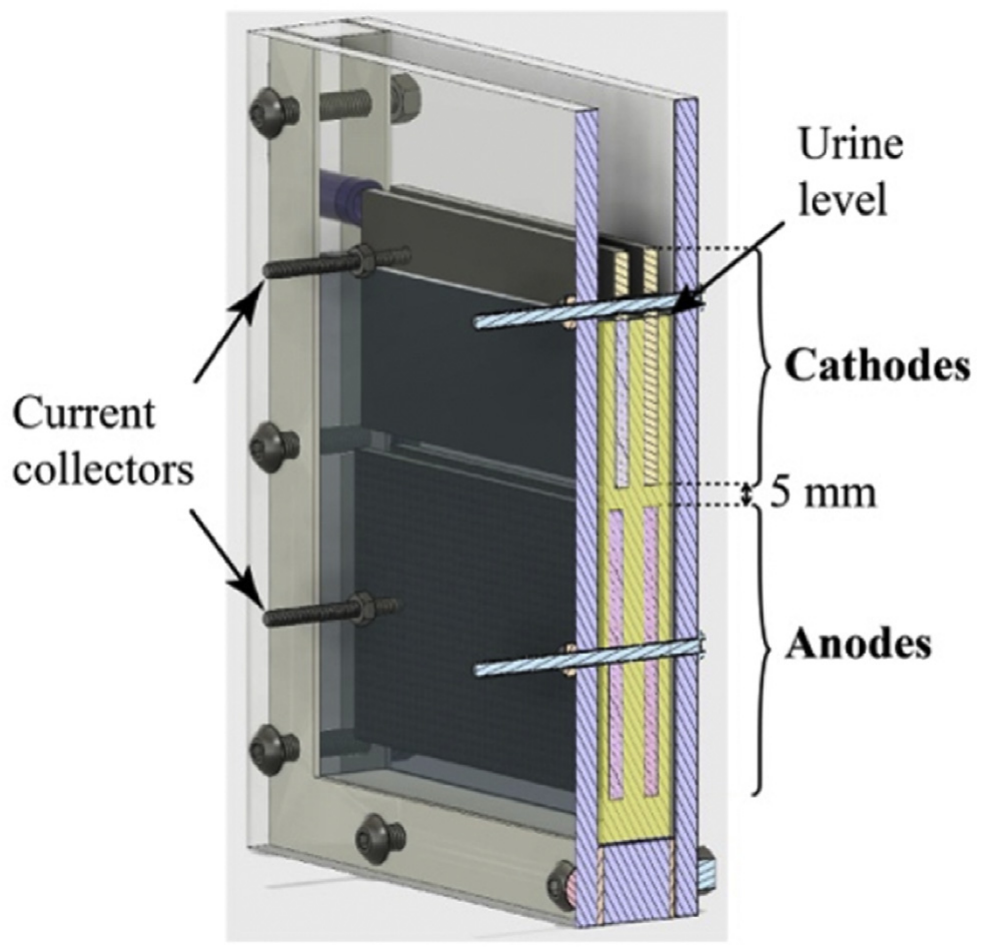
3D representation and section of the SSM-MFC design. Both cathodes and anodes are submerged in the same electrolyte.

The SSM-MFCs were built around a 15 mm acrylic U-shaped core that was sandwiched between two 5 mm thick acrylic plates (Fig. 1). Two U-shaped silicon gaskets maintained the water-tightness. Each SSM-MFCs contained two cathodes placed above two anodes. Two bolts were passing through the whole embodiment and each electrode pairs (Fig. 1). These stainless-steel 316 M4-size bolts were maintaining the electrodes in position and maintaining a good physical contact with them, whilst protruding 20 mm from the sides. Crocodile clips were used to connect the monitoring cables and the fixed resistive loads. The cathodes consisted of a AC/PTFE (activated carbon (AC); polytetrafluoroethylene (PTFE)) mixture pressed on a stainless-steel 316 mesh (8x8 mesh; 8.3 ± 0.2 g; MeshDirect, UK). The mixture was based on 80%wt of AC (SK1 P75, CPL Carbon Link, UK) and 20%wt PTFE (60% dispersion JX-301, Beijing Starget Chemicals Co.,Ltd, China). The final weight of a single cathode was of 16.7 ± 0.5 g with a AC/PTFE mixture loading of 186 ± 7 mg.cm^−2^. Each cathode had the following dimensions: 100 mm long, 45 mm wide and 2 mm thick. In parallel, each anode was fabricated using 1000 cm^2^ carbon fibre veil (10 g.m^−2^; Technical Fibre Products Ltd, Cumbria, UK) that was folded down to a projected surface area of 50 cm^2^ (50 mm × 100 mm). A strip of stainless-steel 316 mesh (100 mm × 15 mm) was fixed in the middle of each anode to act as both a current collector and a structural support.

All the electrodes were submerged in the same electrolyte that was undiluted human urine. The urine was daily collected from a tank pooling together the urine donated by anonymous and healthy individuals with no known previous medical conditions. The urine was kept in a tank for a maximum of 24 h and had probably undergone partial urea hydrolysis by the naturally occurring microflora. The confirmation of this partial hydrolysis was given by the pH values that were well above neutral measuring between 8.5 and 9.3. The solution conductivity of the urine had measured values of 28 ± 2 mScm^−1^. It was previously reported that human urine on average comprises 4.7–10.4 g.L^−1^ dry matter of which 65–85% are organic compounds [11]. Urea is the main constituent of the total organic solids (≈50%) [44]. The SSM-MFCs were inoculated with a mixture comprising 50% (v/v) of the output stream of a matured MFC fuelled with urine, and 50% (v/v) of freshly collected urine. SSM-MFCs with identical height were build and four different immersion depths were investigated. The depths were defined as the proportion of cathode height submerged in the electrolyte: 14, 24, 34 and 44. For the 44 condition, the urine level was 2 mm above the cathode. Each of these depths was tested in duplicate and all error bars indicated in the manuscript (unless otherwise stated) stand for the range of these duplicates. The SSM-MFCs were continuously fuelled by the same multichannel peristaltic pump (Watson & Marlow LTD, UK) and the hydraulic retention time (HRT) was similar among the cells investigated measuring 265 ± 13 min. The SSM-MFCs were initially run under a constant 500 Ω load for ≈5 days, then 300 Ω, and finally 150 Ω. Only the optimum depth was then tested further under a 120 Ω load.

### Data capture and system characterisation

The SSM-MFC outputs were measured in millivolts (mV) against time using an Agilent LXI 34972A data acquisition/switch unit (Farnell, UK). Measurements were recorded every 3 min. The current *I* in Amperes (A) was calculated using Ohm's law, *I* = *V/R*, where *V* is the measured voltage in Volts (V) and *R* is the known value of the external resistor. The power output *P* in watts (W) was calculated as *P* = *I* × *V*.

The initial cathode polarizations were performed in neutral electrolyte media and particularly in phosphate buffer with a pH of 7.06 and a solution conductivity of 14.86 mS.cm^−1^). Before starting the polarisation curve in “clean” media, the cathodes were exposed to the electrolyte for at least 24 h. Linear sweep voltammetry (LSV) were performed using a Biologic potentiostat (SP-50, Science instrument, France) under a three-electrodes configuration with the anodes being used as counter electrodes, the cathodes as the working electrodes and Ag/AgCl (3M KCl) as reference electrode. The scan rate used was 0.25 mV.s^−1^ in order to avoid overestimation of the performance [45]. The cathodes were scanned from open circuit potential (OCP) to −250 mV (vs Ag/AgCl).

The polarisation curves of the mature SSM-MFCs were performed with a two-electrode configuration (potentiostat Biologic SP-50) whereby the reference electrode channel was short-circuited with the counter electrode that was connected to the cathode and the working electrode channel was connected to the anode. Simultaneously a reference electrode (Ag/AgCl) was placed equidistant between anode and cathode (i.e. 5 mm from each other, Fig. 1) and the anode and cathode separate potential variation during the polarisation curve was measured using a PicoTech data logger (ADC-24, Pico Technology Ltd). This operation was done in order to obtain the polarisation curves of both anodes and cathodes separately. Also in this case, the scan rate was 0.25 mV.s^−1^ and ranged from OCV to 50 mV.

The chemical oxygen demand (COD) analyses were performed using the potassium dichromate oxidation method (COD HR test vials, Camlab, UK) with 0.2 mL of inlet and outlet samples taken before and after the MFC treatment. The duplicates samples of the inlet were taken in the tubbing commonly feeding all the SSM-MFCs. The outlet samples were taken directly at the output of each SSM-MFCs. The outputs of the conditions tested in duplicate were then averaged. The results here presented show the average of two distinct SSM-MFCs having the same immersion height. The vials were heated at 150 °C during two hours and cooled to room temperature before the measurements were taken using an MD 200 photometer (Lovibond, UK).

## Results and discussion

### Cathodes polarisation curves in clean media

**Fig. 2:**
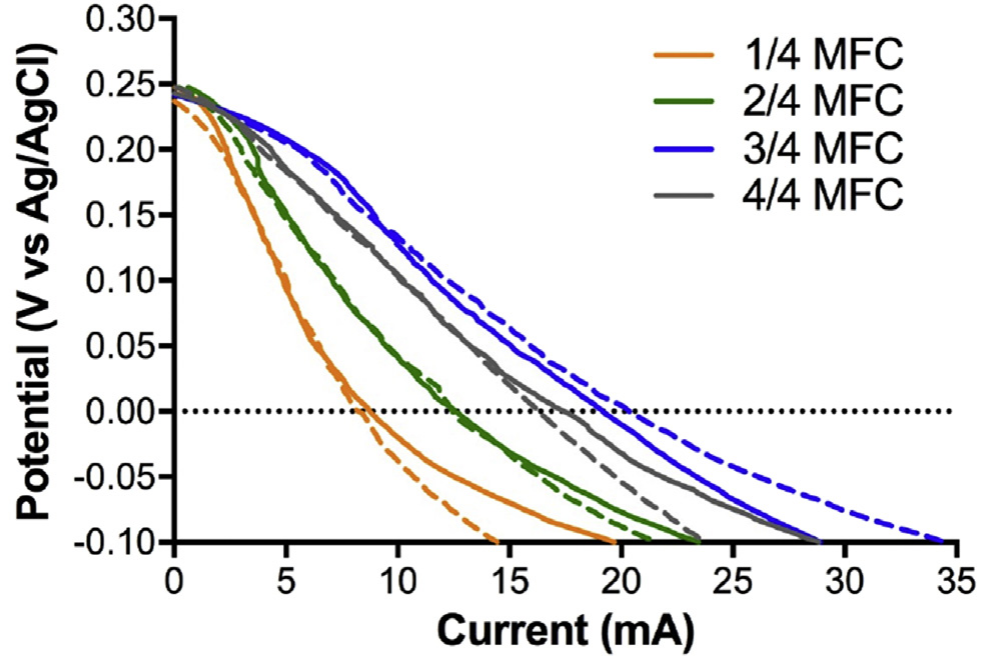
Polarisation curves of the cathodes in phosphate buffer (pH = 7.06; EC = 14.86 mS.cm^−1^) prior inoculation. Solid and dashed lines represent duplicate independent reactors. The data are not presented as average with error bars in order to emphasise the reproducibility of the tests, at low current levels.

The cathodes were initially characterised in “clean” media (i.e. phosphate buffer solution, pH = 7.06; EC = 14.86 mS.cm^−1^) and using a Ag/AgCl reference electrode to run the actual MFC reactor under a three-electrode configuration. The hypothesis being that the increase of the urine column height, implies more cathode surface area in contact with the liquid electrolyte, therefore the oxygen reduction reaction (ORR) rate should be enhanced. Interestingly, Fig. 2 shows that the initial open circuit potential levels (OCP) of the eight cathodic conditions investigated, were similar and consistent, measuring 250 ± 5 mV vs Ag/AgCl. Considering that the theoretical potential value of the ORR at pH = 7 is roughly +600 mV vs Ag/AgCl (3M KCl), the activation overpotential levels measured were approximately 355 mV. These results are in agreement with previous literature utilising activated carbon based cathodes [46], [47].

The results from the polarisation curves show an increase in electrocatalytic activity when the submerged part increase from 14, 24 to 34 of the cathode height. Particularly, considering the current produced at 0 mV vs Ag/AgCl, the cathode submerged 14 produced 8.44 ± 0.4 mA. In comparison, the current increased up to 12.5 ± 0.1 mA when under 24 conditions, and to 19.7 ± 0.8 mA when under 34 condition. This corresponded to an enhancement of 48% and 133%, respectively. Interestingly the electrochemical output slightly decreased when the cathodes were fully submerged (44 condition) even if remain better than the 24 condition measuring a produced current of 16.8 ± 0.8 mA (Fig. 2). The latter corresponded to a reduction of 15% compared to the most performing condition (i.e. 34). Overall, the results show that the cathodes behaviours are reproducible.

At this initial stage and in “clean” media, results indicate that the optimum immersion height was 34 of the cathode submerged in the electrolyte (Fig. 2). The more the surface area of the cathode is submerged into the electrolyte, the higher are the performance (Fig. 2). However, results of these polarisation curves already show that in clean media the cathodes is limited when completely submerged (i.e. 44 condition; Fig. 2). This might be due to the fact that the despite higher surface area was in contact with the liquid electrolyte, the oxygen diffusion from the surface of the water column (air/liquid interface) to the bottom part of the cathode might be limiting.

### Temporal power production under continuous loading

**Fig. 3:**
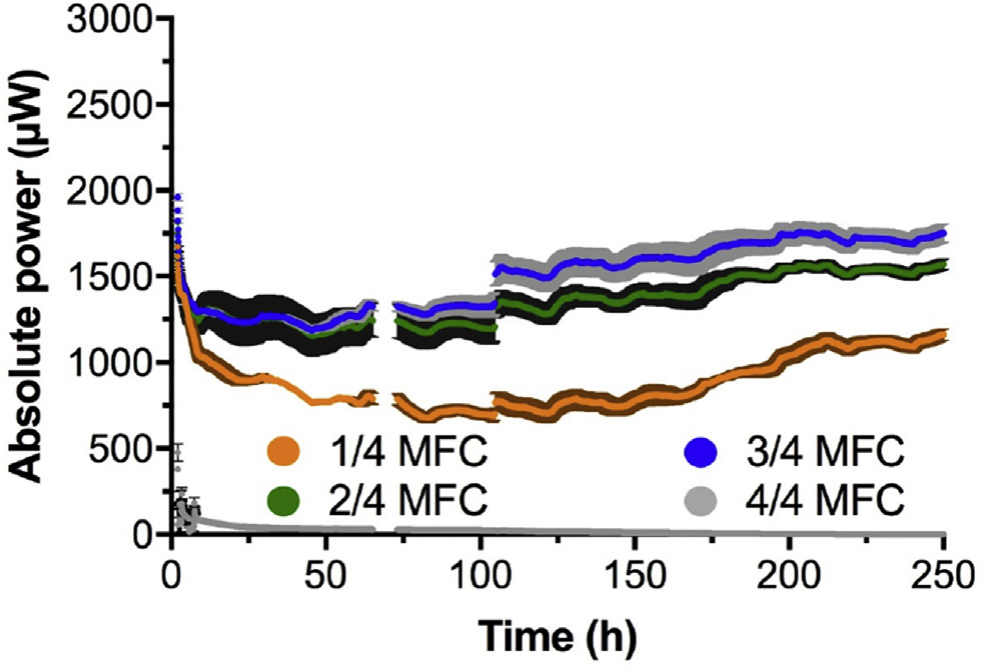
Temporal power evolution under 150Ω, starting 7 days after inoculation. The gap in the data was due to a power failure in the lab and the step change (∼100^th^ hour) was due to a polarisation run.

After the cathode polarisation curves, the SSM-MFCs were inoculated using the effluent of other MFCs also fuelled with urine and fresh urine with a ratio in volume of 1:1. Microbial fuel cells were left in open circuit for roughly 5 h prior connecting the external load. Following the initial phase of inoculation, the resistive loads applied to the SSM-MFCs were progressively decreased from 500 Ω (≈5 days), 300 Ω (≈2 days) to 150 Ω (10 days). Since the SSM-MFCs were placed under continuous flow of fuel, once the produced power plateaued, it was considered that the SSM-MFCs reached steady state. Once the steady states were reached, then heavier loads were applied. An example of this condition (steady state) is clearly visible in Fig. 3, after 150 h when placed under the 150 Ω constant load.

The results recorded for 10 days under continuous 150 Ω external load show that the 24 and 34 immersion conditions had the highest power output compared to the other two conditions that were investigated. The SSM-MFCs under 34 condition were the most efficient in agreement with the cathode polarisation results obtained in phosphate buffer. The SSM-MFCs that had their cathodes fully submerged (44 conditions) did not produce any significant power (3.0 ± 1.8 μW; Fig. 3). Differently than cathode polarisation experiment carried out in clean media (Fig. 2), the SSM-MFCs with fully submerged cathodes did not have good performances in a complex media colonized by life. This probably indicates that the implication of the microbiota activity (e.g. anoxia). Considering the last 75 h of the experiment, during which steady state was reached (n = 1800; Fig. 3), the SSM-MFCs with the cathodes submerged to 14 were producing ≈38% less power than the SSM-MFCs under 34 conditions (1075 ± 85 μW and 1725 ± 32 μW during the last 75 h, respectively). Compared to the 34 conditions, the SSM-MFCs with 24 submerged cathodes were producing 89% less power (1535 ± 29 μW).

### Polarisation of the SSM-MFCs in urine

After maintaining steady state condition for a period of time of 3 days (Fig. 3), the SSM-MFCs were characterised through an overall polarisation curve in a classic two-electrode configuration, with the electrolyte completely replaced by fresh urine. The anode and cathode potentials were at the same time recorded separately, using another data logger, against a single Ag/AgCl 3M KCl reference electrode, which was placed equidistant from both electrodes. SSM-MFCs were operating at constant electrolyte pH of 9.3. At this pH, the theoretical open circuit voltage (OCV) of an operating MFC is averagely 1130 mV. This is the consequence of the redox potential difference between the cathodic reaction involving the oxygen (≈+460 mV vs Ag/AgCl at pH = 9.3) and the anodic organic oxidation reaction performed by the anaerobic microbiota (≈-670 mV vs Ag/AgCl at pH = 9.3).

**Fig. 4:**
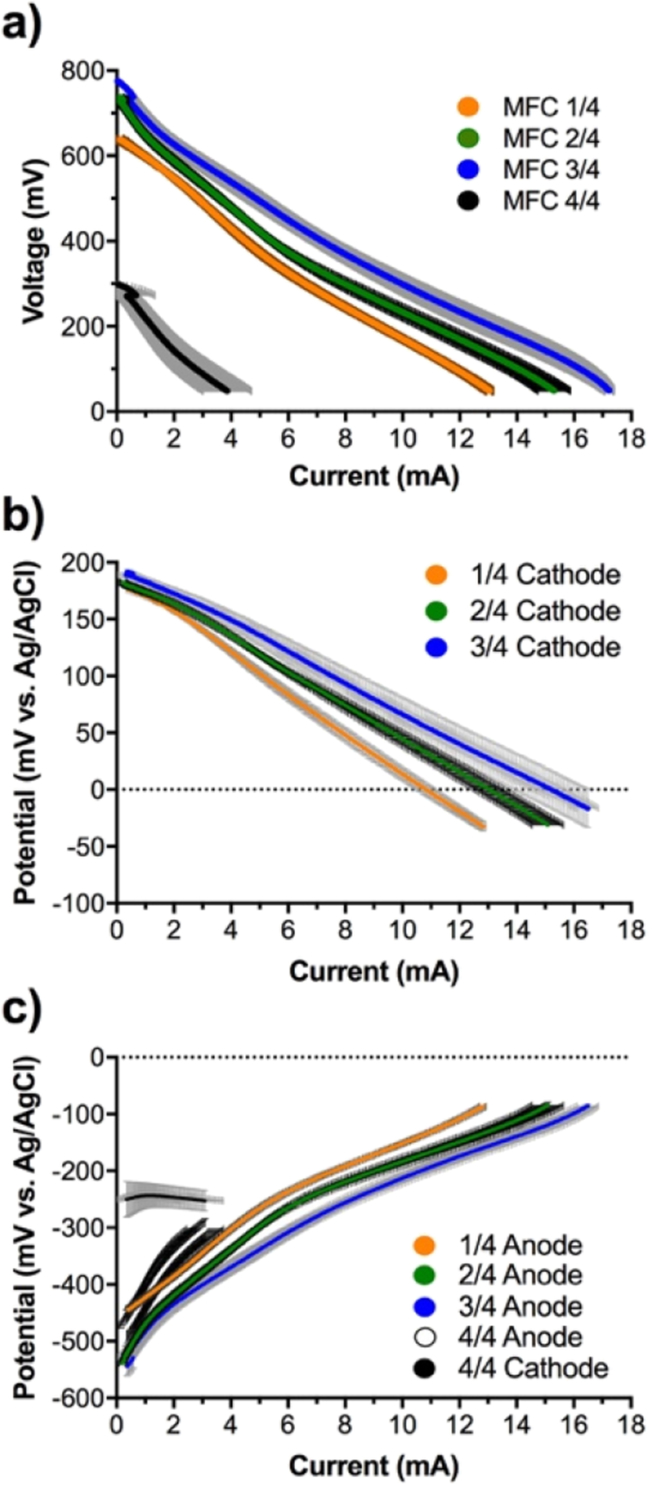
Electrochemical characterisation of the mature SSM-MFCs. a) Voltage-current polarisation curves of the whole cell form OCV to 50 mV. b) Potential-current polarisation curves of the cathodes. c) Potential-current polarisation curves of the anodes. The curves are the average of each conditions duplicates and the error bars stand for the values range. N.B. *The cathode potential-current curve for the fully submerged*44*condition, is shown in the anode graph, due to its negative values.*

As shown by the polarisation curves, beside the SSM-MFCs that had completely submerged cathodes, the SSM-MFCs displayed two OCV levels. On one hand, the 24 and 34 conditions had similar OCV values of 733 ± 7 mV and 755 ± 28 mV, respectively. On the other hand, the SSM-MFC with cathode submerged to 14 had OCV values of 643 ± 7 mV (Fig. 4a). The fully submerged cathodes displayed the lowest OCV values of 237 ± 16 mV. The 34 condition displayed the higher electrochemical performance, followed by the 24 and 14 conditions. The SSM-MFCs with the fully submerged cathodes displayed the worst performance (Fig. 4a).

Generally, the slopes of all semi-submerged cathodes were somewhat linear, implying that they were driven by ohmic losses. However, some differences could be observed. Despite starting from the same OCV, the 34 had higher slope than 24, for lower current, whereas both of them had a similar slope at higher current (>7 mA). The SSM-MFC with cathodes submerged to 14 had similar slope compared to the 34 conditions, even if it seems penalised mainly from the initial starting point that was roughly 110 mV lower (Fig. 4a). This difference of OCV values indicates that a phenomenon is affecting either the cathode or the anode operating in the latter conditions. In order to understand which of the two electrodes is affected, the anodes and cathodes were considered and studied separately.

The cathode potential curves indicate that, with the exception of the fully submerged cathodes, all conditions displayed a similar cathode OCP (Fig. 4b). These results imply that the differences in the measured OCVs were then mainly affected by the anode OCP and not by the cathode OCP. Averagely, the cathode OCP values of all the semi-submerged cathodes were 183 ± 8 mV vs Ag/AgCl. These are values that are in agreement with previously published literature [35]. Conversely, the OCP of the fully submerged cathodes were displaying a negative value of −250 ± 32 mV vs Ag/AgCl (Fig. 4c). Negative cathode OCP implies that when the cathodes were completely submerged (i.e. urine level 2 mm above the cathode) complete anoxic environment is occurring. This observation confirm that part of the cathode is necessary to be exposed directly to the atmosphere for SSM-MFC to function efficiently [41]. This finding is of extreme importance since it implies that complete anoxia is reached at 2 mm depth in a urine column colonised by microorganisms. However, as shown by the results, the cathodes of all the semi-submerged were clearly not in anoxia, even though they were submerged more than 2 mm in depth. It be could hypothesized that the emerged part of these cathodes was acting as a redox snorkel for the biofilm developing in the anaerobic part of the urine column by driving the potential of the cathodes as if they were completely in aerobic conditions. This hypothesis is supported by previous studies that employ this “snorkel phenomenon” to enhance chemical oxygen demand (COD) removal rate in wastewater [48], [49]. The SSM-MFCs having 34 submerged cathodes had the best electrochemical performance (Fig. 4b), followed by the SSM-MFCs having 24 submerged cathodes, and finally the SSM-MFCs having 14 submerged cathodes. This might be due to the fact that more surface area of the electrode is directly in contact to the electrolyte and therefore more active sites are available for catalysing and reducing oxygen. Moreover, the ohmic resistance decreased with more cathode area exposed to the electrolyte and this is confirmed by the decreasing slope of the cathodes (Fig. 4b).

Considering the anode potential curves, the anode OCP of the 24 and 34 conditions had similar values of −539 ± 4 mV vs Ag/AgCl and −544 ± 18 mV vs Ag/AgCl, respectively (Fig. 4c). These values are comparable to previously published work in slightly alkaline environment and anaerobic conditions [50]. Conversely, the SSM-MFCs having 14 submerged cathodes had higher anode OCP values of −445 ± 6 mV vs Ag/AgCl. This higher anode OCPs could be explained by the lower column height of urine that separate the anode electrode from the atmosphere and consequently it can be speculated that that oxygen or other oxidants (e.g. nitrate) could interfere with the anodic oxidation potential. This result, is further supported by the slope of the 14 submerged SSM-MFC being the same as the anode potential of the 24 submerged cathode (Fig. 4c). However, another hypothesis could be that the anode potential reflects the limitation of the cathode potential due to the fact that less surface area of the electrode is exposed to the electrolyte. In comparison to these two tested conditions, the anode potential curve of the 34 conditions is flatter and display the best electrochemical performance, in accordance with previous results (Fig. 3, Fig. 4a,b).

### Power production and treatment efficiency

An important parameter to consider for practical implementation is the maximum power that can be produced by a microbial fuel cell (MFC) along with its treatment efficiency. When deployed under real condition of use, most MFC systems try to find a balance between these two aspects [51], [52], [53]. Hence, with the exception of microbial electrochemical snorkel [48], [49], microbial fuel cells do not run under maximum current but they run close to the maximum power transfer point.

**Fig. 5:**
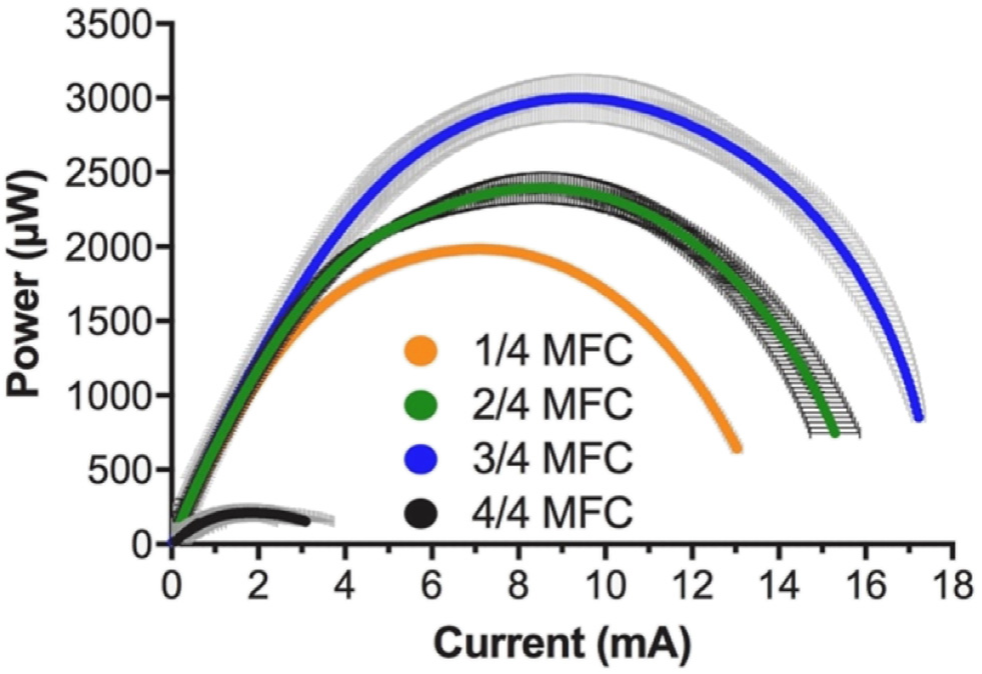
Electrochemical characterisation of the mature SSM-MFCs. Current-power polarisation curves of the whole. The curves are the average of each conditions duplicates and the error bars stand for the values range.

The power curves were obtained from the polarisation curves shown in Fig. 4a. Power curves results indicate that when the cathodes were completely submerged, the SSM-MFCs produce low levels power of 211 ± 55 μW (Fig. 5). Conversely, results from the power curves shows that, the SSM-MFC having 34 submerged cathodes produced the highest power level of 3000 ± 157 μW at a voltage of 321 ± 1 mV and a current of 9.4 ± 0.5 mA (Fig. 5). In terms of maximum power, the following best conditions were the SSM-MFCs obtained with 24 submerged cathodes (2396 ± 101 μW; 281 ± 1 mV; 8.5 ± 0.4 mA) and then with 14 submerged cathodes (1984 ± 21 μW; 281 ± 1 mV; 7.1 ± 0.1 mA) (Fig. 5). Interestingly, if the two-latter tested conditions had different power levels, their maximum power transfer point was reached at the same voltage (281 ± 1 mV). Submerging the cathodes from 14 to 24 increased the potential maximum power production of 21%. A further 14 increase of the submerged cathode surface area enhanced the maximum power production of a further 25%, resulting in a total increase of 51%. Finally, completely submerging the cathodes decreased the potential maximum power production by 89%. These results confirm that the more the surface area of cathode is immersed, the higher the maximum potential power produced was, whilst maintaining the need of having part of the cathodes exposed to air.

As explained above, deploying MFC-system in real life environment implies the treatment of organic waste streams. Measuring the chemical oxygen demand (COD) removal is the most common way to evaluate the treatment efficiency of a system. Therefore, the COD was measured at the inlet of the SMM-MFCs and compared to the values measured at the outlet of the four tested conditions. It has to be reminded that COD removal rates are correlated to the hydraulic retention time (HRT) and that during the whole experiment, the HRT between the tested conditions were kept very similar as explained above.

**Fig. 6:**
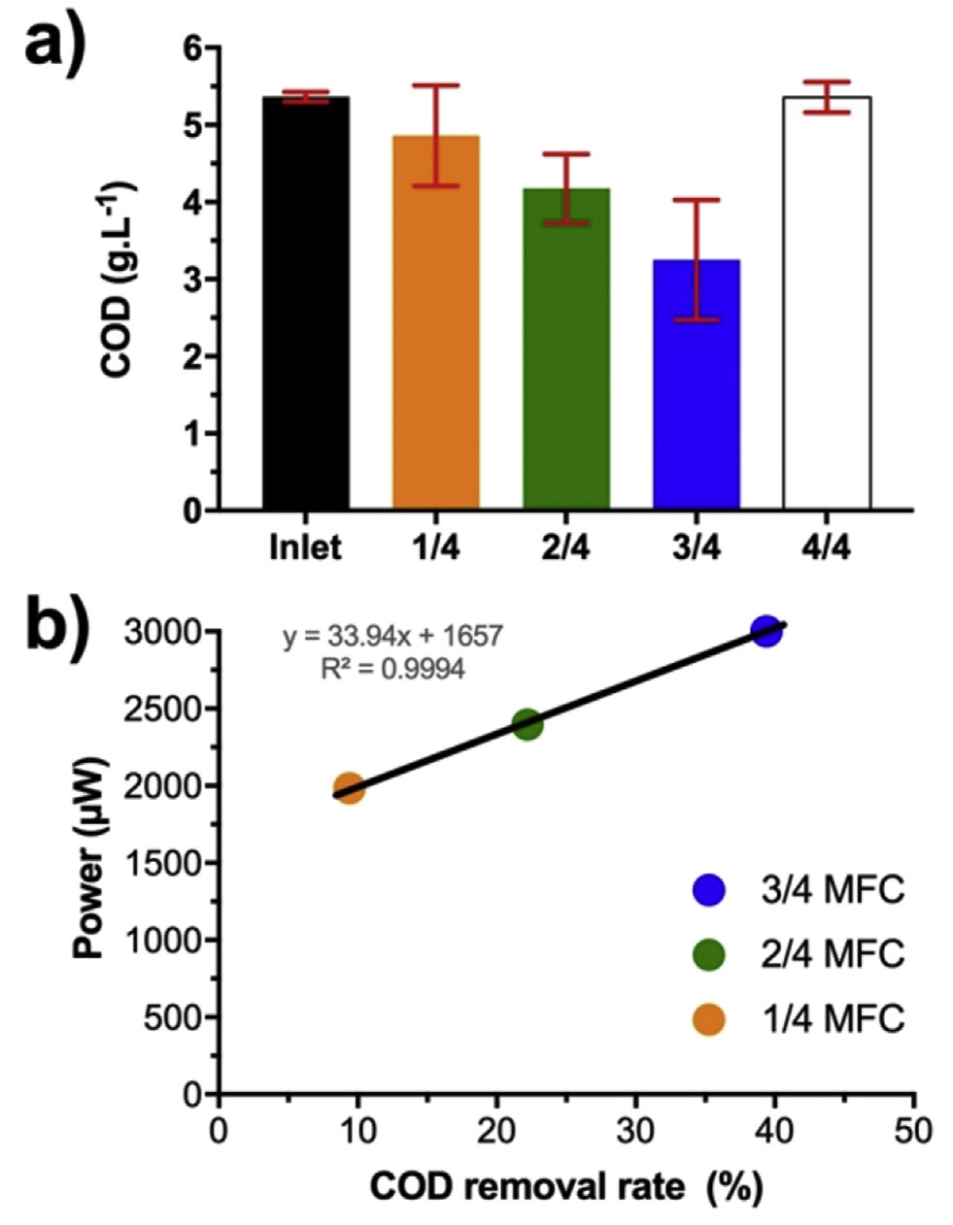
(a) Chemical oxygen demand (COD) concentration between the inlet common to all the SSM-MFCs and the respective outlets of each SSM-MFC. The data are the average from duplicate and independent SSM-MFCs and the error bars stand for the values range. (b) Plot of the average power against the COD removal rate depending on the tested conditions.

The SSM-MFCs had an initial COD loading of 5.37 ± 0.06 g_COD_.L^−1^ (Fig. 6a). Compared to the tested three semi-submerged cathode conditions, the SSM-MFCs with fully submerged cathode displayed an insignificant COD removal of ≈0.1%. Conversely, the semi-submerged cathodes displayed an organic load removal ranging from 9.4%, 22.2%, to 39.4%, in correlation to the cathode immersion height of 14 , 24 , and 34 respectively (Fig. 6a). The final COD concentration for the three condition was 4.86 ± 0.65 g_COD_.L^−1^, 4.18 ± 0.45 g_COD_.L^−1^ and 3.25 ± 0.78 g_COD_.L^−1^ respectively.

In order to see a correlation between power and treatment efficiency, the average maximum power produced (Fig. 5) was plotted against the COD removal rate (Fig. 6a). Results show that the COD removal rate was positively correlated to the power/current produced and therefore to the immersion height of the cathodes (i.e. surface area of cathode in contact with the electrolyte) (Fig. 6b). These results demonstrate that the SSM-MFCs having 34 submerged cathodes were the most efficient in terms of COD removal.

### Stability of the system over time

The long-term stability of microbial fuel cells is often not reported in literature but it is of extreme importance to evaluate possible practical application and evaluate the robustness of the system over time. The most performant SSM-MFCs were therefore monitored over a period of 30 days to assess the stability of the SSM-MFC system. Based on the results previously described, the SSM-MFCs with 34 submerged cathodes that were the best performing were selected. Following results from the polarisation experiment (Fig. 5), the resistive load was changed to 120Ω and the voltage monitored over time (sampling 1 point every 3min). The power was then calculated using ohms' law (*P* = *V* × *I*).

**Fig. 7:**
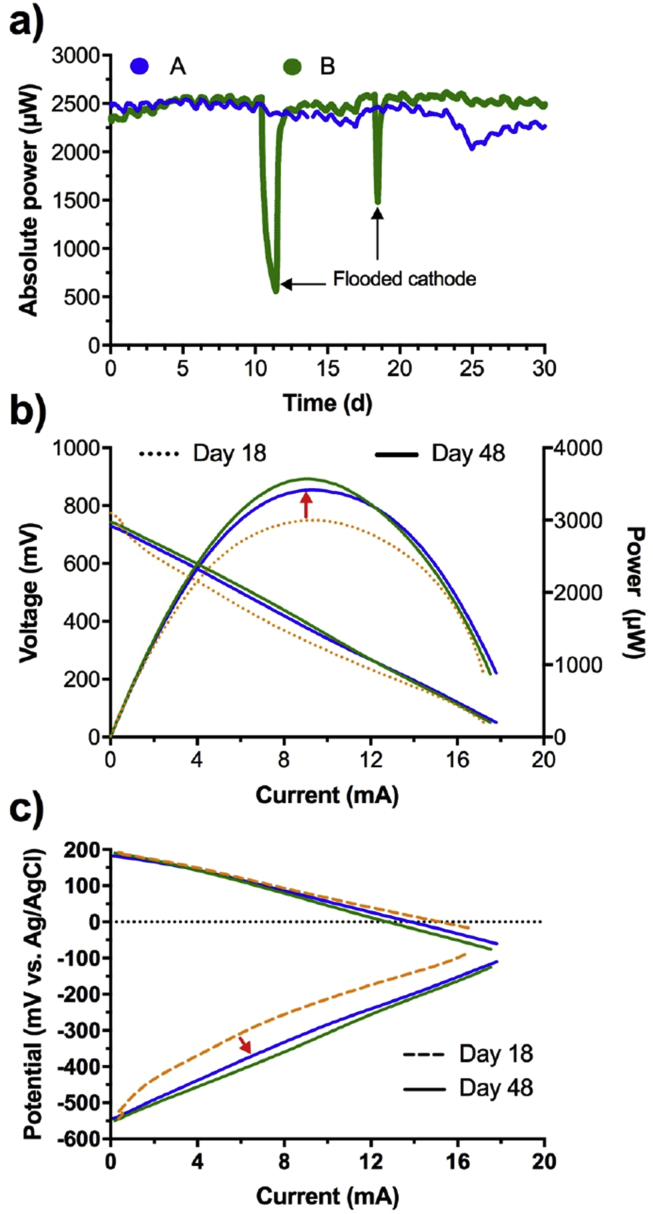
Performance of the duplicate SSM-MFCs having 34 submerged cathodes (green and blue). a) Temporal evolution of the power produced by both duplicate over a 30 days' period. Electrochemical characterisation of the cells after these 30 days with the current-power polarisation curves of the whole cell (b) and the current-potential polarisation of each electrodes (c). The orange curves are the average performance of the same duplicate prior the 30 days run and are extracted from Fig. 4, Fig. 5 (34 condition). The arrow show the change of trend after 30 days of maturation. (For interpretation of the references to color in this figure legend, the reader is referred to the Web version of this article.)

During the course of the experiment, the outlet pipe of SSM-MFC-B got clogged twice (Fig. 7a, MFC-B, green color). The consequence of a clogged pipe is that the urine level inside the SSM-MFC rises and completely submerged the cathodes causing drastic drop in the power output recorded. This behaviour further supports the fact that completely submerged cathodes are detrimental to the performance of SSM-MFCs. Interestingly, the results show that once the pipe was manually unclogged and the level of urine decreased covering only ¾ of the cathode, the MFC-B rapidly recovered its previous power output, thus illustrating the robustness of system toward perturbation (Fig. 7a). Without taking the clogging issue into account, results show that both duplicate had similar power output over 23 days. Over that period of time, the SSM-MFCs were producing 2469 ± 70 μW (n = 21668). From day 23 to day 25, an unidentified phenomenon occurred which affected the power performance of MFC-A. From day 25 to day 30 the power increased but did not reach the same power output as at day 23 (Fig. 7a). The power output can be considered anyway reproducible with an average at day 30 of 2398 ± 116 μW (n = 960). Nonetheless, results show that the setup was stable over a period of 30 days.

At day 30, the SSM-MFCs were then refuelled with fresh urine and left in OCV for 1 h. Overall polarisation curves of the SSM-MFCs were measured with the simultaneous recording of anode and cathode electrode potentials during the polarisation and presented in Fig. 7b and c respectively. Interestingly, the polarisation curves show similar OCV and slope compared to the one run initially. The power obtained actually increased by 16.7% compared to the initial recorded power. The potentials of anode and cathode recorded separately were important to understand the change in performance shown in the overall polarisation curve (Fig. 7b). Cathode polarisation curves measured after 30 days in steady-state operations were similar to the one recorded initially (Fig. 7c). Remarkably, the anode polarisation curves improved over time probably due to a better anode colonisation from electroactive bacteria. The anode and cathode polarisation curves of the SSM-MFCs showed reproducibility with a very low deviation.

## Conclusion

In this study, carbon veil anode and activated carbon-based cathode were investigated in SSM-MFCs fuelled with urine. The main goal of this work was to identify the best cathode submersion height in order to increase the power output and the organic removal of the SSM-MFC. Among the four different heights investigated (14, 24, 34 and 44), the cathode and overall performance increased progressively from 14 to 34 indicating that a greater contact between cathode and electrolyte positively affect the oxygen reduction electrocatalysis. A further increases in height till the cathode was fully submerged, lowered drastically the cathode and cell potential suggesting the establishment of anoxic conditions. One month long-terms performance were run for the SSM-MFCs with the cathode immersed for 34. The power measured through polarisation curves actually indicated an increase of 16.7% from the initial value of 3.0 mW–3.5 mW. Anode and cathode polarisation curves showed that the cathode behaviour was constant over the 1 month operation while the anode actually improved over time.
